# Factors Associated With Low-Value Cancer Screenings in the Veterans Health Administration

**DOI:** 10.1001/jamanetworkopen.2021.30581

**Published:** 2021-10-22

**Authors:** Linnaea Schuttner, Bjarni Haraldsson, Charles Maynard, Christian D. Helfrich, Ashok Reddy, Toral Parikh, Karin M. Nelson, Edwin Wong

**Affiliations:** 1 Health Services Research and Development, VA Puget Sound Health Care System, Seattle, Washington; 2 Department of Medicine, University of Washington, Seattle; 3 Iowa City VA Healthcare System, Iowa City, Iowa; 4 Department of Health Systems and Population Health, University of Washington, Seattle; 5 Geriatrics and Extended Care, VA Puget Sound Healthcare System

## Abstract

**Importance:**

Most clinical practice guidelines recommend stopping cancer screenings when risks exceed benefits, yet low-value screenings persist. The Veterans Health Administration focuses on improving the value and quality of care, using a patient-centered medical home model that may affect cancer screening behavior.

**Objective:**

To understand rates and factors associated with outpatient low-value cancer screenings.

**Design, Setting, and Participants:**

This cohort study assessed the receipt of low-value cancer screening and associated factors among 5 993 010 veterans. Four measures of low-value cancer screening defined by validated recommendations of practices to avoid were constructed using administrative data. Patients with cancer screenings in 2017 at Veterans Health Administration primary care clinics were included. Excluded patients had recent symptoms or historic high-risk diagnoses that may affect test appropriateness (eg, melena preceding colonoscopy). Data were analyzed from December 23, 2019, to June 21, 2021.

**Exposures:**

Receipt of cancer screening test.

**Main Outcomes and Measures:**

Low-value screenings were defined as occurring for average-risk patients outside of guideline-recommended ages or if the 1-year mortality risk estimated using a previously validated score was at least 50%. Factors evaluated in multivariable regression models included patient, clinician, and clinic characteristics and patient-centered medical home domain performance for team-based care, access, and continuity previously developed from administrative and survey data.

**Results:**

Of 5 993 010 veterans (mean [SD] age, 63.1 [16.8] years; 5 496 976 men [91.7%]; 1 027 836 non-Hispanic Black [17.2%] and 4 539 341 non-Hispanic White [75.7%] race and ethnicity) enrolled in primary care, 903 612 of 4 647 479 men of average risk (19.4%) underwent prostate cancer screening; 299 765 of 5 770 622 patients of average risk (5.2%) underwent colorectal cancer screening; 21 930 of 469 045 women of average risk (4.7%) underwent breast cancer screening; and 65 511 of 458 086 women of average risk (14.3%) underwent cervical cancer screening. Of patients screened, low-value testing was rare for 3 cancers, with receipt of a low-value test in 633 of 21 930 of women screened for breast cancer (2.9%), 630 of 65 511 of women screened for cervical cancer (1.0%), and 6790 of 299 765 of patients screened for colorectal cancer (2.3%). However, 350 705 of 4 647 479 of screened men (7.5%) received a low-value prostate cancer test. Patient race and ethnicity, sociodemographic factors, and illness burden were significantly associated with likelihood of receipt of low-value tests among screened patients. No single patient-, clinician-, or clinic-level factor explained the receipt of a low-value test across cancer screening cohorts.

**Conclusions and Relevance:**

This large cohort study found that low-value breast, cervical, and colorectal cancer screenings were rare in the Veterans Health Administration, but more than one-third of patients screened for prostate cancer were tested outside of clinical practice guidelines. Guideline-discordant care has quality implications and is not consistently explained by associated multilevel factors.

## Key Points

**Question:**

What rates and factors are associated with low-value screenings (outside clinical guidelines) for common cancers in the Veterans Health Administration?

**Findings:**

In this cohort study of 5 993 010 veterans, less than 3% of cancer screening recipients received a low-value test for breast, cervical, or colorectal cancer, but 39% of men screened for prostate cancer received a low-value test. No single factor (patient, clinician, clinical, or organizational characteristic) was associated with low-value screening receipt across these 4 cancer types.

**Meaning:**

Findings suggest that low-value prostate cancer screenings are common in the Veterans Health Administration, although factors associated with low-value screenings differ by cancer type.

## Teaser

This cohort study assesses the rates and factors associated with outpatient low-value cancer screenings among nearly 6 million veterans who visited Veterans Health Administration primary clinics.

## Introduction

Health care without benefit or in which the potential harm outweighs the benefit is considered low value. Cancer screening can become low value, for example, with increasing age, greater illness burden, or lower life expectancy^1,2^; in these scenarios, short-term risks (such as procedural complications or testing burden) outweigh the expected benefits from detecting slow-growing cancers.^3,4,5^ Many clinical practice and organizational guidelines recommend stopping cancer screenings when life expectancy falls below a threshold, such as 10 years. Despite guidelines and relative risks, more than 50% of adults with reduced life expectancy report ongoing cancer screening.^6,7,8^ Inappropriate screening is also costly; Medicare spent $790 million on guideline-discordant prostate, cervical, and colon cancer tests in 2009.^9^

Multifactorial influences may affect cancer screenings. One conceptual model describes hierarchical factors associated with cancer care delivery.^10^ Patient sociodemographic factors, insurance, and more mutable elements (eg, attitudes) may be influential.^7,11,12^ Clinician- and team-based associated factors include communication and cultural norms but also roles, teamwork, and staffing. Systems-level organizational structures, community resources, and state policies also appear relevant.^13,14,15,16^ Despite growing understanding, it is unclear how multilevel factors are associated with low-value cancer screenings, particularly within an integrated health system with salaried clinicians, such as the Veterans Health Administration (VHA).

The VHA uses a multidisciplinary patient-centered medical home (PCMH) model in more than 900 clinics, emphasizing teamwork, population health management, continuity, and care quality. This model also increased capacity for preventative screenings through care registries, expanded staffing, and dedicated infrastructure.^17^ Although PCMH models have been associated with improved preventative care delivery,^18,19^ connections to low-value cancer screenings have not been examined. Paradoxically, more low-value screenings could occur with expanded access or protocolized screenings delivered via enhanced team structures.^17^ Alternatively, low-value cancer screenings may decrease with greater clinician continuity from PCMH implementation^17^; another study found that continuity was associated with fewer low-value tests.^20^

Understanding of low-value cancer screenings is evolving.^8,9,21,22,23^ Adding to emerging evidence, validated recommendations on low-value screenings have been published.^24^ Examining rates and factors associated with low-value cancer screenings within the VHA using these recommendations may provide evidence for interventions targeting the appropriateness of care or the development of low-value performance measures similar to Medicare’s Merit-based Incentive Payment System.^25^ Clarifying how low-value screenings are associated with PCMH organizational characteristics may inform future intervention design or implementation strategies. We sought to describe the prevalence and association of multilevel factors, including key PCMH domains, with 4 common low-value cancer screenings (breast, cervical, colorectal, and prostate) within the VHA.

## Methods

### Study Overview and Data Sources

We developed operational definitions of low-value screenings for each cancer (Table 1^8,9,21,22,26,27^) from validated recommendations of ambulatory practices to avoid.^24^ Those recommendations define low-value screening as outside clinical practice guidelines among patients exceeding age recommendations or with reduced life expectancy. We assessed whether patients undergoing cancer screenings had a low-value test and associated factors, among each cohort. This analysis was designated quality improvement rather than research as part of VHA primary care evaluation efforts, and thus it was not subject to institutional review board approval or waiver and was exempt from the requirement to obtain patient consent. This study follows the Strengthening the Reporting of Observational Studies in Epidemiology (STROBE) reporting guideline for cohort studies.^28^

**Table 1.** Definitions of Low-Value Outpatient Cancer Screenings

Term	Breast	Cervical	Colorectal	Prostate
Numerator (ie, low-value screening)	Average-risk females <40 y or LE <1 y	Average-risk females <21 y, >65 y with prior adequate screenings, or with prior hysterectomy^a^	Average-risk adults <50 y or LE <1 y	Average-risk males, age <50 y, >69 y, or LE <1 y
Denominator	Average-risk females >18 y with screening mammography	Average-risk females >18 y with screening Pap or high-risk HPV testing	Average-risk adults >18 y with screening colonoscopy, sigmoidoscopy, or fecal occult home test	Average-risk males >18 y with screening PSA
Repeated procedure logic	Included only if no prior mammography in prior 11 mo (presumed repeat was diagnostic)	Pap and HPV occurring on separate dates within a 90-d window counted as single index event in FY 2017	Only most recent screen during 12 mo of FY 2017 (repeat presumed owing to incomplete colonoscopy)	Only most recent PSA included during 12 mo of FY 2017
Exclusions (from both numerator and denominator)	Family history of breast cancer, personal history of genetic carrier risk, breast cancer, breast mass, or received radiation in last 10 y	HIV/AIDS, history of exposure to diethylstilbestrol before birth, abnormal Pap smear, or cervical cancer in last 10 y	Personal history of colectomy; colorectal cancer; colon polyps; inflammatory bowel disease; or family history of colorectal cancer in last 14 y. Gastrointestinal symptoms in 12 mo prior to index screening	African American race or ethnicity;^b^ family history of prostate cancer; personal history of prostate cancer in prior 10 y. Urinary or prostate-related symptoms in 90 d prior to index PSA

Abbreviations: FY 2017, fiscal year 2017 (October 1, 2016, to September 30, 2017); HPV, human papillomavirus; LE, life expectancy; Pap, Papanicolaou test; PSA, prostate-specific antigen.

^a^ Prior adequate screenings defined as 2 prior negative Pap plus HPV tests within 10 years, with at least 1 test in last 5 years; or 3 prior negative Pap tests within 10 years, with the most recent test in the last 3 years; or 2 prior HPV tests within 10 years, with the most recent test in the last 5 years.

^b^ Cohort excluded non-Hispanic Black patients a priori because clinical practice guidelines recommended individualizing screening at younger ages (40-54 years) owing to higher prostate cancer risk.

We constructed 4 cohorts and examined multilevel factors using VHA administrative data: encounter data, patient and clinician demographic characteristics, and facility covariates from the Corporate Data Warehouse^29^; staffing covariates from the Provider Specialty Workforce Report; clinic locations from the Site Tracking System; and county-level descriptors linked by patient zip code to Area Health Resource Files.^30^ Race and ethnicity were defined using algorithms prioritizing self-identification.^31^

### Cohort Criteria and Separate Low-Value Cancer Screening Outcomes

Included patients had at least 1 visit to VHA primary care in 2017 (fiscal year [FY], October 1, 2016, to September 30, 2017). We excluded patients at high risk for cancer (eg, by family history) or with potential diagnostic indications for testing, such as recent symptoms, that might indicate testing was performed for screening. Screening receipt was based on outpatient *Current Procedural Terminology* testing codes for each cancer type, based on prior literature.^9,21,22,26,32,33^ We grouped patients into separate cohorts defined by cancer type, permitting patients to be part of multiple cohorts. Inclusion and exclusion criteria were adapted from prior literature and are summarized in Table 1 (details in eTable 1 in the Supplement). For patients with multiple tests for a cancer (such as fecal immunochemical test and colonoscopy) in FY 2017, we used only 1 index test according to the logic presented in Table 1.^8,9,21,26^ Specific to VHA, prostate cancer screening may persist beyond guideline-recommended age thresholds for older patients owing to higher prostate cancer risk among veterans exposed to Agent Orange, a carcinogenic tactical herbicide predominant in the Vietnam war.^34^ Therefore, we conducted a subgroup analysis of Vietnam-era veterans (≥18 years by May 7, 1975), including this exposure as a possible patient-level factor.

For the breast cancer screening cohort, we included women who had a mammogram, excluding those with a mammogram in 11 months prior to the index test to reduce the likelihood that diagnostic imaging followed a screening test.^26^ Any patient with a high-risk diagnosis, such as breast cancer, in the prior 10 years was also excluded.^9,26,32,33^

For the cervical cancer screening cohort, we included women who underwent a human papillomavirus test or Papanicolaou test, excluding those without an adequate screening history or high-risk diagnoses (eg, abnormal Papanicolaou test) in the prior 10 years^9,21,27,35^ by using a validated algorithm.^21^ For the colorectal cancer screening cohort, we included patients who underwent colonoscopy, sigmoidoscopy, or fecal occult blood testing or fecal immunochemical testing, excluding those with gastrointestinal symptoms in 12 months prior to the index test or with high-risk diagnoses within 14 years prior.^9,22^

For the prostate cancer screening, we included men who underwent a prostate-specific antigen test, excluding those with genitourinary symptoms for 90 days prior or high-risk diagnoses in the last 10 years (eg, prostate cancer).^8,9^ We also excluded African American men in accordance with other low-value literature^24^ and practice guidelines recommending individualized screening decisions for African American men aged 40 to 54 years given higher prostate cancer risk.^36^ We acknowledge that our use of constructed categories of race and ethnicity necessitates interpretation of our findings within the context of ancestral, structural, cultural, socioeconomic, and other factors not represented in our study.

### Measurement of Low-Value Cancer Screenings

Within cohorts, we defined binary outcomes denoting whether testing met low-value criteria (Table 1). We estimated reduced life expectancy using a 1-year probability of mortality of 50% or higher, based on a validated VHA score^37^ applied in prior research.^22^

### Potential Multilevel Factors Associated With Low-Value Care

Potential factors associated with low-value cancer screenings were based on previous studies, conceptual models, and data availability.^8,9,10,16,21,26,38^ Patient factors included sex (colorectal only); race and ethnicity (as 3 categories: non-Hispanic White; non-Hispanic Black; and all others, including Hispanic, American Indian, Alaskan Native, Asian, Pacific Islander, multiracial, Native Hawaiian, 23 additional ethnicity categories, and missing race data^31^); copayment status; Gagne comorbidity score^39^ (≥2 as high); and frailty (JEN Frailty Index,^40^ ≥3 as frail); county-level median household income; and proportion of county residents 25 years or older with high school diplomas. Veterans with a high service-connected disability rating (having conditions considered >50% disabling) or with annual incomes below qualifying thresholds are VHA copayment exempt^41^; copayments have been used as a measure of higher individual socioeconomic status.^42^ Variables were measured at the time of index screenings, except for comorbidity and frailty, which were recorded in the last quarter of FY 2016 to avoid contamination arising from FY 2017 test results. In the prostate cancer subgroup analysis, we also added a binary marker for Agent Orange exposure.

Ordering clinician factors included degree (physician vs other advanced degree); age; sex; primary care clinician status (ie, if clinicians were also the assigned primary care clinician); and full-time equivalent time in clinical care. Clinician factors were recorded during the quarter of FY 2017 when the index test occurred.

Factors describing patients’ outpatient clinic included geographic region; clinic type (community or hospital-based); academic affiliation; urban or rural location; primary care clinician full-time equivalent per clinic (ie, size); complexity level (determined by facility capabilities such as the presence of an intensive care unit); and mean patient panel size per primary care clinician (adjusted for physician vs advance practice clinician). Clinic variables were measured during the final quarter of FY 2016 to assess site-level factors that may have been associated with activities leading up to the index test.

Clinical factors also included 3 composite measures denoting the extent of implementation of PCMH domains, drawn from previously validated measures: team-based care, access to care, and continuity.^18^ These composite measures were constructed from VHA administrative and patient survey data as standardized *z* scores, with higher values denoting better performance. Composite measures were categorized to compare the top quartile of implementing clinics vs lower performers for each domain.

### Statistical Analysis

We described unadjusted rates of low-value screenings for each cancer type. We applied exploratory logistic regression models to assess associations of multilevel factors with receipt of low-value testing among patients screened. Models excluded encounters missing patient (7%-15%) or clinic (4%-15%) covariates. We first assessed the mean estimated probability of receiving a low-value test using the marginal standardization method by sequentially adding patient-, clinic-, and clinician-level factors. To assess independent associations from factors controlling for the others, we conducted multivariable models analyzing levels simultaneously in 2 stages owing to high proportions of missing clinician data (18%-82%). Primary models included patient and clinic factors; secondary models added clinician factors and excluded encounters missing these data. Standard errors are heteroskedastic robust (Huber-White) and account for clustering within clinics. Statistical significance (α) was .05 for exploratory models. Analysis was at the patient level, using Stata, version 16.1 (StataCorp) and R, versions 3.6.2-4.0.4 (R Foundation for Statistical Computing) software.^43,44^ Data were analyzed from December 23, 2019, to June 21, 2021.

## Results

A total of 5 993 010 VHA patients were enrolleed in primary care in 2017 (mean [SD] age, 63.1 [16.8] years; 5 496 976 men [91.7%] and 496 012 women [8.3%]; 1 027 836 non-Hispanic Black [17.2%] and 4 539 341 non-Hispanic White [75.7%] race and ethnicity). For breast cancer, 469 045 women were average risk and 21 930 were screened (1.4%); 633 tests were low value (0.1% of average risk, 2.9% of screened women). For cervical cancer, 458 086 women were average risk and 65 511 were screened (14.3%); 630 tests were low value (0.1% of average risk, 1.0% of screened women). For colorectal cancer, 5 770 622 patients were average risk and 299 765 were screened (5.2%); 6790 tests were low value (0.1% of average risk, 2.9% of screened patients). For prostate cancer, 4 647 479 men were average risk and 903 612 were screened (19.4%); 350 705 tests were low value (7.6% of average risk, 38.8% of screened men). Among cancer screening recipients, most patients were non-Hispanic White males (Table 2) who visited urban hospital-affiliated clinics in the US Southeast (eTable 2 in the Supplement).

**Table 2.** Demographic Characteristics Among Veterans Receiving an Outpatient Cancer Screening in 2017

Characteristic	Veterans, No. (%)			
	Breast	Cervical	Colorectal	Prostate
Patients, No.	21 930	65 511	299 765	903 612
Sex				
Female	21 930 (100)	65 511 (100)	18 834 (6.3)	0
Male	0	0	280 931 (93.7)	903 612 (100)
Age, mean (SD), y	54.9 (9.1)	44.0 (12.5)	63.5 (8.4)	65.8 (9.6)
Race and ethnicity				
White, non-Hispanic	12 067 (55.0)	35 594 (54.3)	219 633 (73.3)	864 576 (95.7)
Black, non-Hispanic	8050 (36.7)	23 244 (35.5)	58 937 (19.7)	NA^a^
Hispanic, other, or unknown^b^	1813 (8.3)	6673 (10.2)	21 195 (7.1)	39 036 (4.3)
US Region				
West	4621 (21.1)	12 909 (19.7)	63 472 (22.7)	172 772 (19.5)
Midwest	1507 (6.9)	9802 (15.0)	55 268 (19.8)	215 949 (24.4)
Northeast	4611 (21.0)	14 851 (22.7)	45 701 (16.3)	172 492 (19.5)
Southeast	11 191 (51.0)	27 949 (42.7)	115 462 (41.3)	325 591 (36.7)
High Gagne score (>2)	1621 (7.4)	3091 (4.7)	23 915 (8.0)	85 326 (9.5)
High frailty score (JFI >3)	12 342 (56.5)	32 393 (49.6)	138 004 (46.2)	447 250 (50.0)
Copay (higher income)	896 (4.3)	2190 (3.9)	18 395 (7.0)	34 410 (10.6)
HS diploma holders (by county), mean (SD), %^c^	58.0 (4.6)	57.6 (4.9)	58.1 (5.4)	58.9 (5.6)
Household income by county, median (SD), $ × 1000	58.4 (13.7)	58.7 (14.5)	56.2 (14.1)	56.5 (14.0)

Abbreviations: HS, high school; JFI, JEN Frailty Index; NA, not applicable.

^a^ Cohort excluded non-Hispanic Black patients a priori because clinical practice guidelines recommended individualizing screening at younger ages (40-54 years) owing to higher prostate cancer risk.

^b^ Category includes Hispanic or American Indian, Alaskan Native, Asian, Pacific Islander, multiracial, Native Hawaiian, or additional 23 race and ethnicities,^31^ and those missing race data.

^c^ Mean proportion of adults 25 years or older with a high school diploma within patient’s county of residence.

### Multivariable Models

In multivariable models, patient factors contributed the greatest proportion of variance to the probability of a received cancer screening being a low-value test. Adjusting for patient factors, the mean estimated probability of receiving a low-value test among patients screened was 38.1% (95% CI, 37.3%-38.9%) for prostate cancer and less than 3% for the other cancer types (breast: 2.8% [95% CI, 2.4-3.3]; cervical: 1.1% [95% CI, 0.8-1.4]; colorectal: 2.2% [95% CI, 2.0-2.4]). In further adjustment, clinician and clinical factors were associated with less than 0.6% of the variation in estimated probability of receiving a low-value test (Table 3).

**Table 3.** Probability of Receipt of a Low-Value Cancer Test Among Screened Patients, Adjusting for Multilevel Factors

Adjustment	Estimated probability, mean (95% CI)^a^			
	Breast	Cervical	Colorectal	Prostate
Patient factors	2.8 (2.4-3.3)	1.1 (0.8-1.4)	2.2 (2.0-2.4)	38.1 (37.3-38.9)
Plus clinic or organization	2.8 (2.4-3.2)	1.2 (0.9-1.4)	2.2 (2.0-2.4)	38.2 (37.4-39.0)
Plus clinician^b^	2.2 (1.9-2.6)	0.8 (0.6-1.0)	1.7 (1.5-1.9)	37.8 (36.9-38.6)

^a^ With cluster-robust standard errors.

^b^ Ordering clinician factors were added last to the models owing to higher proportion of data missingness.

### Patient and Clinical Factors

In models including both patient and clinical characteristics (Figure), among patients screened for breast cancer, those with greater comorbidity (vs low comorbidity: odds ratio [OR], 0.59 [95% CI, 0.38-0.91]; *P* = .02), frailty (vs less frail: OR, 0.72 [95% CI, 0.60-0.86]; *P* < .001), or copays (vs exempt: OR, 0.34 [95% CI, 0.15-0.80]; *P* = .01) were less likely to receive low-value tests. Attending top performing clinics for care continuity resulted in lower odds of receiving a low-value test (vs lower performers: OR, 0.46 [95% CI, 0.27-0.78]; *P* = .004).

**Figure.**
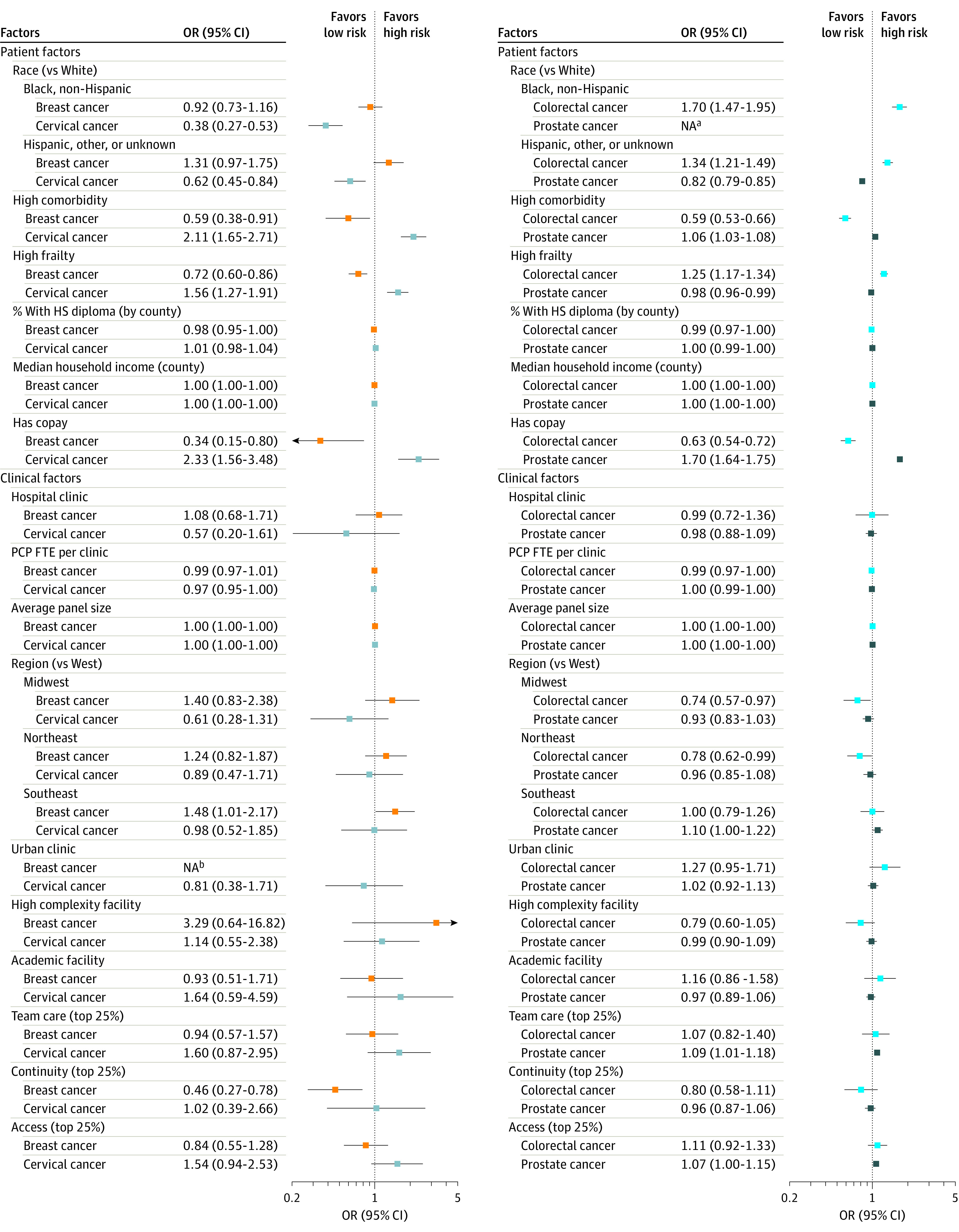
Patient and Clinic Factors Associated With Receipt of Low-Value Test Among Patients Screened for Cancer Clinic performance measures for team-based care, continuity, and access compare top quartile clinics with lower scoring clinics. FTE indicates full-time equivalent; HS, high school; NA, not applicable; OR, odds ratio; and PCP, primary care clinician. ^a^Prostate models excluded Black non-Hispanic patients a priori owing to higher cancer risk. ^b^Breast models dropped urban vs rural owing to nonvariance.

Among patients screened for cervical cancer, compared with non-Hispanic White patients, non-Hispanic Black patients (OR, 0.38 [95% CI, 0.27-0.53]; *P* < .001) and patients who were Hispanic or other races or ethnicities (OR, 0.62 [95% CI, 0.45-0.84]; *P* = .002) were less likely to receive low-value tests. Patients with higher comorbidity burden (OR, 2.11 [95% CI, 1.65-2.71]; *P* < .001), frailty (OR, 1.56 [95% CI, 1.27-1.92]; *P* < .001), or copays (OR, 2.33 [95% CI, 1.56-3.48]; *P* < .001) were more likely to receive low-value tests.

Among patients screened for colorectal cancer, patients who were non-Hispanic Black (OR, 1.70 [95% CI, 1.47-1.95]; *P* < .001) or Hispanic or other races and ethnicities (OR, 1.34 [95% CI, 1.21-1.49]; *P* < .001) were more likely to receive low-value tests than non-Hispanic White patients. Those with higher comorbidity (OR, 0.59 [95% CI, 0.53-0.66]; *P* < .001) and copays (OR, 0.63 [95% CI, 0.54-0.72]; *P* < .001) were less likely, but frailer patients were more likely to receive low-value tests (OR, 1.25 [95% CI, 1.17-1.34]; *P* < .001).

Among patients screened for prostate cancer, Hispanic and other races and ethnicities were less likely to receive low-value tests (vs non-Hispanic White patients: OR, 0.82 [95% CI, 0.79-0.85]; *P* < .001), as were frailer patients (OR, 0.98 [95% CI, 0.96-0.99; *P* = .004). Patients with greater comorbidity (OR, 1.06 [95% CI, 1.03-1.08]; *P* < .001) and copays (OR, 1.70 [95% CI, 1.64-1.75]; *P* < .001) were more likely to receive low-value tests.

### Patient, Clinical, and Clinician Factors

After also adjusting for differences in ordering clinician, among patients screened for breast cancer, only frailty remained associated with lower odds of receiving low-value testing (OR, 0.67 [95% CI, 0.56-0.81]; *P* < .001). For cervical cancer, non-Hispanic Black women remained less likely to receive low-value tests (OR, 0.33 [95% CI, 0.16-0.66]; *P* = .002). For colorectal cancer, patient race and ethnicity, comorbidity, frailty, and copay status remained significant although those patients visiting urban or low-complexity clinics were more likely to receive low-value tests (urban vs rural: OR, 1.51 [95% CI, 1.05-2.17]; *P* = .03; low vs high-complexity: OR, 0.67 [95% CI, 0.48-0.93]; *P* = .02). No substantive differences emerged for prostate cancer screening between models (eTable 3 in the Supplement).

### Prostate Cancer Subgroup Analysis

Among Vietnam-era veterans screened for prostate cancer, 233 314 men had Agent Orange exposure. Adjusting for other patient and clinical factors among men screened for prostate cancer, exposure was associated with lower odds of low-value testing compared with those patients without exposure (OR, 0.95 [95% CI, 0.92-0.99]; *P* = .01).

## Discussion

Our study is among the first to operationalize validated recommendations for low-value testing among common cancer screenings and to examine associations with multilevel factors for the VHA. Overall, testing for breast, colorectal or cervical cancer was rarely low value, among both all average-risk patients or screening recipients. However, low-value prostate cancer tests were more common, received by 7.6% of all average-risk men and 38.8% of screened men. Predominantly patient factors were associated with higher likelihood of receipt of low-value cancer testing among screened patients; however, no single factor was significant in one direction across all 4 cohorts. There was also no clear association between select domains of the VHA PCMH model and low-value test receipt.

The integrated system of the VHA may attract different patient populations and lead to variation in screening activities due to more equitable access, informational continuity, fewer financial barriers to care, or less incentives for services compared with other systems.^45^ In illustration, in contrast to cancer screening disparities in other systems,^46,47,48^ racial and ethnic minority populations have greater parity in the VHA and the US Department of Defense health care systems.^49,50,51^ Patient race and ethnicity, illness burden, and copay status were significantly associated with likelihood of low-value test receipt across cancer screening cohorts, but the direction of association differed. One explanation may relate to patterns of VHA use and testing logistics. Prior research has found that patients with racial and ethnic minority backgrounds, less favorable sociodemographic characteristics, and fewer comorbidities are more reliant on the VHA relative to Medicare for health care services.^45^ Algorithmic protocols for home colorectal cancer screening, standard in the VHA, could lead to more low-value testing in the populations who receive more care within the VHA. Low-value cervical or breast cancer screening may occur infrequently given fewer women veterans compared with men in the VHA, with factors differing by test procedure.^52^ With rare on-site mammography facilities,^53^ breast cancer screening off-site referrals may be particularly burdensome to women with more comorbidities, leading to less frequent low-value testing. By contrast, low-value cervical cancer testing may occur as a byproduct of frequent clinic visits for women with higher comorbidities.

We found high rates of guideline-discordant VHA prostate cancer tests among screened patients. Similar to our findings, other literature has described correlations between prostate-specific antigen testing and overall intensity of health care, with concentrations in affluent, White populations.^8,54,55^ Unlike other cancer screenings in which logistics may be more influential, serology-based prostate-specific antigen tests are easily obtained with other blood tests.^55^ Cancer screenings may depend on patient preference, which may lead to testing outside of clinical guidelines.^56^ Within VHA, we were concerned that persistent testing outside guidelines may be associated with history of Vietnam-era Agent Orange exposure, a risk factor for prostate cancer.^34^ However, a dedicated subgroup analysis did not show that this exposure was associated with more low-value testing.

Few PCMH domains were consistently associated with low-value test receipt for any of the 4 cancers among screened patients. An association between less overall low-value care and better continuity has been described,^20^ but implications for the VHA PCMH model are limited given the isolation of our findings to women screened for breast cancer.

A distinction from prior work is how we measured low-value cancer screening rates. We examined proportions of low-value testing among cancer screening recipients; other studies measured low-value cancer screening among patients at risk for low-value testing (eg, older adults).^8,9,26,57,58^ For example, in 1 investigation, nearly 8% of Medicare beneficiaries older than 75 years received low-value colorectal cancer screening, whereas low-value cervical cancer screening occurred in 7% of female beneficiaries older than 65 years.^9^ Another study of Medicare-enrolled women with life expectancies less than 1 year found that 18% of them still received breast cancer screening.^26^ A VHA study among veterans older than 75 years estimated that 18% recently received low-value prostate cancer testing.^8^ Our low-value testing rates among screened patients are partly explained by denominator demographic factors (eg, most VHA female patients are younger than 65 years,^59^ so less low-value testing occurs among cervical cancer screening recipients). Few comparable studies have examined low-value care among younger individuals (eg, prostate cancer screening in men younger than 50 years). Variation across studies also emphasizes the importance of standardizing low-value definitions.

While in aggregate, low-value cancer screenings may pose greater risk than benefit, testing outside established recommendations must be individualized, as algorithmic decisions may misclassify patients otherwise appropriate for screening.^60^ Clinicians should engage in shared decision-making with those patients. Communication strategies, decision aides, and conceptual frameworks for these challenging conversations have been proposed, supported by the American Cancer Society and the US Preventive Services Task Force.^3,7,61,62^ More sophisticated predictions for who might benefit from screenings, beyond chronologic age, would also help determine screening appropriateness.^63,64,65^ Individualized recommendations for cancer screening may help to advance care quality, particularly for patients with advanced age or poor health status.

### Strengths and Limitations

Defining and measuring low-value care has inherent limitations, particularly reliance on clinical practice guidelines and imperfect life expectancy predictions. A major strength of this study is our approach building from validated recommendations describing cancer screenings unlikely to improve mortality when applied to asymptomatic patients.^24^ Research is needed to define the sensitivity and specificity of our claims-based measures although similar VHA-based approaches show high specificity compared with manual medical record review.^22^ We did not examine factors associated with low-value testing among all eligible patients, which may make our findings more difficult to interpret. We also did not include all cancer screening factors, such as encounter time, individual attitudes, or patient request.^12,56^ We also note aspects that may limit external generalizability, including specific exclusion criteria, data missingness, and studying veterans in a national integrated health system.

## Conclusions

This cohort study found that for patients who were screened, prostate cancer testing frequently occurs outside clinical practice guidelines, including for patients who may not benefit owing to reduced life expectancy. Testing for other common cancers was rarely low value. Several noteworthy patient factors were associated with receipt of low-value cancer screenings among patients tested, but few other multilevel factors were relevant.

## Notes

Supplement. **eTable 1.** Detailed Inclusion/Exclusion Criteria **eTable 2.** Unadjusted Associations of Low-Value Cancer Test Receipt (LVC) Among Those Screened With Patient, Clinic/Organizational, and Clinician-Level Factors **eTable 3.** Multivariable Models With Patient, Clinic, and Clinician-Level Factors Associated With Low-Value Cancer Test Receipt Among Those Screened
