# Supplementary material for: Factors Associated With Low-Value Cancer Screenings in the Veterans Health Administration
Source: JAMA Netw Open. 2021 Oct 22;4(10):e2130581. doi: 10.1001/jamanetworkopen.2021.30581 (PMC8536952; doi:10.1001/jamanetworkopen.2021.30581)
Supplement: Supplement. — eTable 1. Detailed Inclusion/Exclusion Criteria eTable 2. Unadjusted Associations of Low-Value Cancer Test Receipt (LVC) Among Those Screened With Patient, Clinic/Organizational, and Clinician-Level Factors eTable 3. Multivariable Models With Patient, Clinic, and Clinician-Level Factors Associated With Low-Value Cancer Test Receipt Among Those Screened [file jamanetwopen-e2130581-s001.pdf]

Supplementary Online Content

Schuttner L, Haraldsson B, Maynard C, et al. Factors associated with low-value cancer screenings in the Veterans Health Administration. *JAMA Netw Open*. 2021;4(10):e2130581. doi:10.1001/jamanetworkopen.2021.30581

eTable 1. Detailed Inclusion/Exclusion Criteria
eTable 2. Unadjusted Associations of Low-Value Cancer Test Receipt (LVC) Among Those Screened With Patient, Clinic/Organizational, and Clinician-Level Factors
eTable 3. Multivariable Models With Patient, Clinic, and Clinician-Level Factors Associated With Low-Value Cancer Test Receipt Among Those Screened

This supplementary material has been provided by the authors to give readers additional information about their work.

© 2021 Schuttner L et al. *JAMA Network Open*.

eTable 1. Detailed Inclusion/Exclusion Criteria	
**Breast cancer**	**Description**
Inclusions	Mammography (other, diagnostic, screening, unspecified)
Exclusion: patients with prior screening mammography in 11 months	Mammography (screening or diagnostic)
Exclusion: High-risk symptoms/signs in prior 10 years	Breast cancer or history of breast cancer; Carcinoma-in-situ of breast; Breast mass; Genetic risk carrier or family history of high genetic risk; Family history of breast cancer; History of irradiation
**Cervical cancer**	**Description**
Inclusions	Screening PAP; Screening cervical cancer exam; HPV screening
High-risk or other exclusions for patients in past 10 years	Female genital organ cancer, or personal history of genital organ cancer; Benign neoplasm of genital organ; Carcinoma-in-situ of genital organ; Cervical dysplasia; Abnormal PAP; Cervical HPV test positive; Incomplete PAP; HIV/AIDs; DES exposure; Hysterectomy or acquired absence of cervix
**Colon cancer**	**Description**
Inclusions	Fecal immunochemical test; Fecal occult blood test; Colonoscopy; Sigmoidoscopy
Symptom/procedure exclusion codes. Excluded for 12 months prior to index event.	Abdominal swelling, mass, or distension; absent bowel sounds; post-hemorrhagic anemia; colitis; angiodysplasia with hemorrhage; anorexia; aphasia, inguinal hernia; vomiting; bowel habit change; constipation; Crohn's disease; diarrhea; diverticulitis; dysphagia; epigastric pain, mass, or swelling; eructation; fecal abnormalities or incontinence; flatulence or gas pain; gastrointestinal bleeding; heartburn; hematemesis; hyperactive bowel sounds; ileus; inguinal hernia; colon polyps; intussusception; iron deficiency anemia; irritable bowel syndrome; megacolon; melena; nausea; abnormal bowel sounds; other GI abnormalities; other specific signs or symptoms of GI system; ulcerative colitis; history of colon polyps or colorectal cancer; vomiting; underweight or weight loss; visible peristalsis
High-risk exclusions. Excluded for 14 years prior to index event.	Cancer of GI tract or history of GI cancer<br>Colectomy<br>Carcinoma-in-situ of colon or rectum<br>Colon polyps or benign neoplasms of colon or rectum<br>Family history of colon polyps or GI cancer<br>Ulcerative colitis, left sided colitis, or Crohn's disease
**Prostate cancer**	**Description**
Inclusions	PSA testing
Symptom/procedure exclusion codes. Excluded for 90 days prior to index event.	Urinary obstruction or uropathy; Hematuria; Prostatitis, abscess or inflammation; Prostate or male GU organ abnormalities; Weight loss<br>Back pain; Imaging abnormalities of GU organs or musculoskeletal system
High-risk exclusions. Excluded for 10 years prior to index event.	Benign neoplasm of male organs; Carcinoma-in-situ of prostate; Prostate cancer or history of prostate cancer; Elevated PSA; Prostatectomy<br>Androgen deprivation therapy; Family history of GU cancer

© 2021 Schuttner L et al. *JAMA Network Open*.

eTable 2. Unadjusted Associations of Low-Value Cancer Test Receipt (LVC) Among Those Screened With Patient, Clinic/Organizational, and Clinician-Level Factors

Count (%) shown for categorical variables, and mean (SD) for continuous variables, unless otherwise noted.

	Cervical			Breast			Prostate			Colorectal		
	**No LVC**	**LVC (%)**	**Total**	**No LVC**	**LVC (%)**	**Total**	**No LVC**	**LVC (%)**	**Total**	**No LVC**	**LVC (%)**	**Total**
Patient factors												
Sex												
F	64,881 (99.0)	630 (1.0)	65,511 (100)	21,297 (97.1)	633 (2.9)	21,930 (100)	0 (0)	0 (0)	0 (0)	18,010 (6.2)	824 (12.1)	18,834 (6.3)
M	0 (0)	0 (0)	0 (0)	0 (0)	0 (0)	0 (0)	552,907 (61.2)	350,705 (38.8)	903,612 (100)	274,965 (93.9)	5,966 (87.9)	280,931 (93.7)
Age	43.8 (12.3)	69.7 (4.3)	44.0 (12.5)	55.5 (8.5)	35.4 (3.2)	54.9 (9.1)	63.4 (5.6)	69.5 (12.9)	65.8 (9.6)	64.0 (7.7)	42.4 (8.1)	63.5 (8.4)
Race/eth.												
White	35,118 (54.1)	476 (75.6)	35,594 (54.3)	11,726 (55.1)	341 (53.9)	12,067 (55.0)	527,020 (95.3)	337,556 (96.3)	864,576 (95.7)	215,382 (73.5)	4,251 (62.6)	219,633 (73.3)
Black	23,133 (35.7)	111 (17.6)	23,244 (35.5)	7,823 (36.7)	227 (35.9)	8,050 (36.7)	0 (0)	0 (0)	0 (0)	57,015 (19.5)	1,922 (28.3)	58,937 (19.7)
Hisp/ Other/ Unk	6,630 (10.2)	43 (6.8)	6,673 (10.2)	1,748 (8.2)	65 (10.3)	1,813 (8.3)	25,887 (4.7)	13,149 (3.8)	39,036 (4.3)	20,578 (7.0)	617 (9.1)	21,195 (7.1)
Region												
West	12,773 (19.7)	136 (21.6)	12,909 (19.7)	4,508 (21.2)	113 (17.9)	4,621 (21.1)	107,247 (19.8)	65,525 (19.0)	172,772 (19.5)	61,840 (22.6)	1,632 (26.1)	63,472 (22.7)
Midwest	9,715 (15.0)	87 (13.8)	9,802 (15.0)	1,465 (6.9)	42 (6.6)	1,507 (6.9)	134,301 (24.8)	81,648 (23.7)	215,949 (24.4)	54,301 (19.8)	967 (15.5)	55,268 (19.8)
NE	14,708 (22.7)	143 (22.7)	14,851 (22.7)	4,485 (21.1)	126 (19.9)	4,611 (21.0)	106,103 (19.6)	66,389 (19.3)	172,492 (19.5)	44,733 (16.4)	968 (15.5)	45,701 (16.3)
SE	27,685 (42.7)	264 (41.9)	27,949 (42.7)	10839 (50.9)	352 (55.6)	11,191 (51.0)	194,311 (35.9)	131,280 (38.1)	325,591 (36.7)	112,784 (41.2)	2,678 (42.9)	115,462 (41.3)
Gagne												
Low (<2)	61,865 (95.4)	555 (88.10)	62,420 (95.3)	19,700 (92.5)	609 (96.2)	20,309 (92.6)	498,475 (90.5)	316,107 (90.5)	814,582 (90.5)	269,415 (92.0)	6,435 (94.8)	275,850 (92.0)
High (≥2)	3,016 (4.7)	75 (11.9)	3,091 (4.7)	1,597 (7.5)	24 (3.8)	1,621 (7.4)	52,271 (9.5)	33,055 (9.5)	85,326 (9.5)	23,560 (8.0)	355 (5.2)	23,915 (8.0)

© 2021 Schuttner L et al. *JAMA Network Open*.

eTable 2. Unadjusted associations of low-value cancer test receipt (LVC) among those screened with patient, clinic/organizational, and clinician-level factors. Count (%) shown for categorical variables, and mean (SD) for continuous variables, unless otherwise noted.												
	**Cervical**			**Breast**			**Prostate**			**Colorectal**		
	**No LVC**	**LVC (%)**	**Total**	**No LVC**	**LVC (%)**	**Total**	**No LVC**	**LVC (%)**	**Total**	**No LVC**	**LVC (%)**	**Total**
Patient factors (continued)												
Frailty												
Low (< 3)	32,658 (50.5)	224 (35.6)	32,882 (50.4)	9,188 (43.3)	320 (50.6)	9,508 (43.5)	271,227 (49.5)	176,194 (50.8)	170,423 (49.2)	157,201 (53.9)	3,369 (49.9)	160,570 (53.8)
High (≥ 3)	31,988 (49.5)	405 (64.4)	32,393 (49.6)	12,029 (56.7)	313 (49.5)	12,342 (56.5)	276,827 (50.5)	170,423 (49.2)	447,250 (50.0)	134,625 (46.1)	3,379 (50.1)	138,004 (46.2)
Copay												
Exempt	54,160 (96.2)	565 (91.9)	54,725 (96.2)	19,609 (95.7)	591 (98.3)	20,200 (95.8)	491,259 (93.4)	290,698 (89.4)	34,410 (10.6)	240,423 (93.0)	5,486 (95.5)	245,909 (93.0)
Have copay	2,140 (3.8)	50 (8.1)	2,190 (3.9)	886 (4.3)	10 (1.7)	896 (4.3)	34,545 (6.6)	34,410 (10.6)	68,955 (8.1)	18,138 (7.0)	257 (4.5)	18,395 (7.0)
% > 25y w/ HS dipl.[a]	57.6 (4.9)	58.4 (4.8)	57.6 (4.9)	58.0 (4.6)	57.6 (4.6)	58.0 (4.6)	58.9 (5.5)	58.9 (5.7)	58.9 (5.6)	58.1 (5.4)	57.3 (5.5)	58.1 (5.4)
Med. house.[a]	58,697 (14,529)	57,248 (15,005)	58,682 (14,534)	58,352 (13,746)	58,676 (12,699)	58,361 (13,718)	56,425 (13,836)	56,532 (14,244)	56,467 (13,996)	56,200 (14,102)	56,707 (14,128)	56,211 (14,102)
Clinic/organizational factors[b]												
PCP panel size	815.8 (228.7)	850.8 (190.7)	816.1 (228.3)	770.5 (229.9)	777.4 (251.9)	770.7 (230.6)	821.6 (231.0)	831.8 (228.6)	825.6 (230.1)	916.1 (309.7)	904.4 (340.5)	915.8 (310.4)
PCP FTE per clinic	16.4 (9.2)	14.0 (6.3)	16.4 (9.1)	19.4 (9.3)	19.1 (9.6)	19.4 (9.3)	14.7 (9.3)	14.5 (9.2)	14.6 (9.3)	12.8 (10.3)	12.7 (9.7)	12.8 (10.3)
Hospital (VA) or community affiliation												
VA	51,098 (84.4)	489 (79.9)	51,587 (84.3)	15,728 (77.2)	434 (73.4)	16,162 (77.1)	480,874 (88.7)	300,882 (87.3)	781,756 (88.2)	158,623 (58.0)	3,834 (61.4)	162,457 (58.0)
Comm.	9,454 (15.6)	123 (20.1)	9,577 (15.7)	4,649 (22.8)	157 (26.6)	4,806 (22.9)	61,088 (11.3)	43,960 (12.8)	105,048 (11.9)	115,035 (42.0)	2,411 (38.6)	117,446 (42.0)
Clinic location												
Urban	57,573 (95.1)	562 (91.8)	58,135 (95.1)	20,331 (99.8)	591 (100)	20,922 (99.8)	492,648 (90.9)	312,894 (90.7)	805,542 (90.8)	231,759 (84.7)	5,544 (88.8)	237,303 (84.8)
Rural	2,976 (4.9)	50 (8.2)	3,026 (5.0)	46 (0.2)	0 (0)	46 (0.2)	49,314 (9.1)	31,948 (9.3)	81,262 (9.2)	41,738 (15.3)	700 (11.2)	42,438 (15.2)
Facility Complexity												

© 2021 Schuttner L et al. *JAMA Network Open*.

eTable 2. Unadjusted associations of low-value cancer test receipt (LVC) among those screened with patient, clinic/organizational, and clinician-level factors. Count (%) shown for categorical variables, and mean (SD) for continuous variables, unless otherwise noted.												
	**Cervical**			**Breast**			**Prostate**			**Colorectal**		
	**No LVC**	**LVC (%)**	**Total**	**No LVC**	**LVC (%)**	**Total**	**No LVC**	**LVC (%)**	**Total**	**No LVC**	**LVC (%)**	**Total**
Clinic/organizational factors[b] (continued)												
Low	6,495 (10.7)	89 (14.5)	6,584 (10.8)	256 (1.3)	3 (0.5)	259 (1.2)	126,995 (23.4)	82,277 (23.9)	209,272 (23.6)	46,626 (17.0)	1,052 (16.9)	47,678 (17.0)
High	54,057 (89.3)	523 (85.5)	54,580 (89.2)	20,121 (98.7)	588 (99.5)	20,709 (98.8)	414,967 (76.6)	262,565 (76.1)	677,532 (76.4)	227,032 (83.0)	5,193 (83.2)	232,225 (83.0)
Academic affiliation												
No	11,078 (18.3)	127 (20.8)	11,205 (18.3)	4,077 (20.0)	139 (23.5)	4,216 (20.1)	99,568 (18.4)	67,343 (19.5)	166,911 (18.8)	123,821 (45.3)	2,575 (41.2)	126,396 (45.2)
Yes	49,474 (81.7)	485 (79.3)	49,959 (81.7)	16,300 (80.0)	452 (76.5)	16,752 (79.9)	442,394 (81.6)	277,499 (80.5)	719,893 (81.2)	149,837 (54.8)	3,670 (58.8)	153,507 (54.8)
Team-based care domain performance												
Low	54,663 (92.0)	515 (84.6)	55,178 (91.9)	17,573 (86.2)	509 (86.1)	18,082 (86.2)	474,559 (89.4)	299,048 (88.2)	773,607 (89.0)	215,313 (85.5)	4,962 (85.4)	220,275 (85.5)
High	4,764 (8.0)	94 (15.4)	4,858 (8.1)	2,804 (13.8)	82 (13.9)	2,886 (13.8)	56,218 (10.6)	39,840 (11.8)	96,058 (11.1)	36,473 (14.5)	848 (14.6)	37,321 (14.5)
Continuity domain performance												
Low	59,064 (97.8)	587 (95.9)	59,651 (97.8)	20,140 (98.8)	587 (99.3)	20,727 (98.9)	528,010 (98.2)	336,858 (98.1)	864,868 (98.2)	252,340 (92.3)	5,901 (94.8)	258,241 (92.4)
High	1,350 (2.2)	25 (4.1)	1,375 (2.3)	237 (1.2)	4 (0.7)	241 (1.2)	9,451 (1.8)	6,383 (1.9)	15,834 (1.8)	20,971 (7.7)	325 (5.2)	21,296 (7.6)
Access domain performance												
Low	36,719 (60.8)	315 (51.5)	37,034 (60.7)	11,212 (55.0)	357 (60.4)	11,569 (55.2)	318,038 (59.2)	200,596 (58.4)	518,634 (58.9)	188,008 (68.8)	4,339 (68.7)	192,347 (68.8)
High	23,695 (39.2)	297 (48.5)	23,992 (39.3)	9.165 (45.0)	234 (39.6)	9,399 (44.8)	219,423 (40.8)	142,645 (41.6)	362,068 (41.1)	85,303 (31.2)	1,887 (30.3)	87,190 (31.2)
Ordering clinician factors[b]												
Age	51.4 (9.6)	56.0 (8.6)	51.5 (9.6)	50.9 (9.6)	50.8 (9.6)	50.9 (9.6)	53.3 (9.6)	53.8 (9.7)	53.5 (9.7)	53.9 (9.9)	54.8 (9.9)	53.9 (9.9)
FTE (%)	82.1 (28.0)	84.7 (22.2)	82.2 (28.0)	84.0 (27.6)	87.0 (25.5)	84.0 (27.6)	95.5 (16.8)	95.4 (17.3)	95.5 (17.0)	86.7 (25.8)	88.6 (24.4)	86.7 (25.7)
Sex												
M	1,593 (10.1)	5 (5.2)	1,598 (10.1)	2,754 (17.8)	59 (15.6)	2,813 (17.7)	229,751 (47.7)	145,131 (48.1)	374,882 (47.8)	95,699 (48.0)	1,752 (47.5)	97,451 (48.0)

© 2021 Schuttner L et al. *JAMA Network Open*.

eTable 2. Unadjusted associations of low-value cancer test receipt (LVC) among those screened with patient, clinic/organizational, and clinician-level factors. Count (%) shown for categorical variables, and mean (SD) for continuous variables, unless otherwise noted.												
	**Cervical**			**Breast**			**Prostate**			**Colorectal**		
	**No LVC**	**LVC (%)**	**Total**	**No LVC**	**LVC (%)**	**Total**	**No LVC**	**LVC (%)**	**Total**	**No LVC**	**LVC (%)**	**Total**
Ordering clinician factors[b] (continued)												
F	14,125 (89.9)	92 (94.9)	14,217 (89.9)	12,721 (82.2)	319 (84.4)	13,040 (82.3)	238,815 (49.5)	147,039 (48.8)	385,854 (49.2)	103,553 (52.0)	1,937 (52.5)	105,490 (52.0)
Is PCP?												
No	44,795 (69.0)	480 (76.2)	45,275 (69.1)	5,513 (25.9)	244 (38.6)	5,757 (26.3)	70,758 (12.8)	49,092 (14.0)	119,850 (13.3)	92,041 (31.4)	3,033 (44.7)	95,074 (31.7)
Yes	20,086 (31.0)	150 (23.8)	20,236 (30.9)	15,784 (74.1)	389 (61.5)	16,173 (73.8)	482,149 (87.2)	301,613 (86.0)	783,762 (86.7)	200,727 (68.6)	3,750 (55.3)	204,477 (68.3)
Degree												
Non-MD	7,654 (38.7)	76 (51.7)	7,730 (38.8)	3,664 (23.2)	101 (26.0)	3,765 (23.3)	123,024 (25.8)	77,188 (25.9)	200,212 (25.8)	47,812 (23.4)	1,003 (26.5)	48,815 (23.5)
MD	12,134 (61.3)	71 (48.3)	12,205 (61.2)	12,120 (76.8)	288 (74.0)	12,408 (76.7)	354,662 (74.3)	221,232 (74.1)	575,894 (74.2)	156,526 (76.6)	2,789 (73.6)	159,315 (76.6)

[a]County-level: proportion of county with a high school diploma; median household income by county. [b]Clinic and clinician level factors are described at the patient-level.

© 2021 Schuttner L et al. *JAMA Network Open*.

eTable 3. Multivariable Models With Patient, Clinic, and Clinician-Level Factors Associated With Low-Value Cancer Test Receipt Among Those Screened

	Breast				Cervical				Colorectal				Prostate			
Patients	n = 13,747				n = 9,404				n = 148,456				n = 654,869			
	OR	95L	95H	*P*	OR	95L	95H	*P*	OR	95L	95H	*P*	OR	95L	95H	*P*
Race/ethnicity (v. White non-Hisp.)																
Black non-Hisp.	0.99	0.77	1.28	0.97	0.33	0.16	0.66	**<0.01**	2.00	1.70	2.35	**<0.001**	N/A *(a priori* exclusion*)*			
Hisp/Other/Unk	1.27	0.83	1.92	0.27	0.97	0.50	1.87	0.93	1.37	1.18	1.58	**<0.001**	0.83	0.79	0.86	**<0.001**
High Gagne (vs. low)	0.67	0.36	1.24	0.20	1.10	0.40	2.99	0.86	0.42	0.34	0.51	**<0.001**	1.05	1.02	1.07	**<0.001**
High Frailty (vs. low)	0.67	0.56	0.81	**<0.001**	1.40	0.79	2.49	0.25	1.22	1.10	1.34	**<0.001**	0.97	0.96	0.99	**<0.01**
% > 25y w/ HS diploma (county)	0.96	0.94	0.99	**0.01**	1.00	0.95	1.06	0.95	0.98	0.96	1.00	0.06	1.00	1.00	1.01	0.84
Med. househld income (county)	1.00	1.00	1.00	**0.01**	1.00	1.00	1.00	0.25	1.00	1.00	1.00	0.23	1.00	1.00	1.00	0.24
Copay (vs. none)	0.32	0.09	1.09	0.07	2.63	0.81	8.54	0.11	0.67	0.55	0.82	**<0.001**	1.70	1.64	1.77	**<0.001**
VA affiliation (vs. community)	0.97	0.57	1.65	0.90	1.62	0.38	6.89	0.52	0.81	0.57	1.15	0.24	0.99	0.88	1.10	0.81
PCP FTE/clinic	0.98	0.96	1.01	0.16	0.98	0.95	1.02	0.39	0.99	0.97	1.00	0.08	1.00	0.99	1.00	0.21
PCP panel size	1.00	1.00	1.00	0.57	1.00	1.00	1.00	0.61	1.00	1.00	1.00	0.99	1.00	1.00	1.00	**0.01**
Region (vs. Northwest)																
Midwest	1.60	0.81	3.17	0.18	1.30	0.45	3.72	0.63	0.73	0.52	1.03	0.07	0.96	0.86	1.08	0.50
Northeast	1.15	0.69	1.90	0.60	1.29	0.45	3.73	0.64	0.78	0.58	1.05	0.10	0.99	0.87	1.12	0.85
Southeast	1.70	0.98	2.96	0.06	1.52	0.61	3.74	0.37	0.97	0.70	1.35	0.86	1.15	1.05	1.26	**0.003**
Urban (vs. rural)	-	-	-	-	0.93	0.36	2.35	0.87	1.51	1.05	2.17	**0.03**	1.03	0.93	1.15	0.54
High complex. facility	-	-	-	-	1.50	0.60	3.75	0.39	0.67	0.48	0.93	**0.02**	0.99	0.90	1.10	0.92
Acad. affiliation	1.07	0.55	2.06	0.84	0.45	0.11	1.88	0.27	1.02	0.71	1.46	0.90	0.97	0.88	1.07	0.56

© 2021 Schuttner L et al. *JAMA Network Open*.

	Breast				Cervical				Colorectal				Prostate			
Team-based care (Top vs. lower)	1.01	0.60	1.70	0.96	1.59	0.75	3.39	0.23	1.17	0.86	1.60	0.33	1.09	1.00	1.18	**0.04**
Continuity (Top vs. lower)	0.91	0.54	1.55	0.74	0.39	0.09	1.76	0.22	0.90	0.64	1.26	0.53	0.92	0.84	1.01	0.10
Access (Top vs. lower)	0.93	0.59	1.47	0.75	1.53	0.66	3.54	0.32	1.17	0.92	1.49	0.21	1.07	1.00	1.15	0.07
Prov. Age	1.00	0.99	1.01	0.86	1.03	0.99	1.07	0.11	1.01	1.00	1.02	0.01	1.00	1.00	1.01	**<0.001**
Prov. As PCP	-	-	-	-	-	-	-	**-**	1.47	1.02	2.12	**0.04**	**-**	-	-	-
Prov. Sex	1.22	0.95	1.58	0.12	1.95	0.59	6.41	0.27	1.04	0.86	1.25	0.70	1.01	0.98	1.05	0.45
Prov. FTE	1.62	0.88	2.98	0.12	0.89	0.31	2.61	0.84	1.41	0.91	2.18	0.12	0.94	0.85	1.03	0.16
Prov. As physician	0.99	0.71	1.40	0.97	1.30	0.67	2.50	0.44	0.84	0.69	1.04	0.10	0.95	0.90	1.01	0.09

(-) Noted where model omitted covariate due to non-variance.

© 2021 Schuttner L et al. *JAMA Network Open*.
